# Work environment and health in the Norwegian fishing fleet - a field study on board deep-sea fishing vessels

**DOI:** 10.1186/2046-7648-4-S1-A48

**Published:** 2015-09-14

**Authors:** Mariann Sandsund, Erik U Høye, Cecilie T Heidelberg, Lisbeth Aasmoe

**Affiliations:** 1 SINTEF Technology and Society, Department of Health Research, Trondheim, Norway; 2 University Hospital of North Norway, Department of Occupational and Environmental Medicine, Tromsø, Norway; 3 Norwegian University of Science and Technology, Department of Biology, Trondheim, Norway; 4 The Arctic University of Norway, Department of Medical Biology, Tromsø, Norway

## Introduction

Workers in the Norwegian fishing fleet who are exposed to unfavorable conditions such as cold, noise, heavy lifting, inconvenient working hours, long work days and excessive strain, are liable to be negatively affected in terms of their health and work performance. Such conditions may also affect thermoregulatory responses, thermal sensation and comfort. There is a lack of knowledge regarding interaction between work, the work environment and the working health of fishermen. The objective of this study was to identify work strain and cold-related problems in deep-sea fishing vessel crew members.

## Methods

Field studies were performed on board five deep-sea fishing vessels in the Norwegian Sea between March 2014 and February 2015. One hundred and fifty crew members completed a questionnaire covering work-related health topics, and detailed subjective perceptions of their thermal work environment. In addition, physiological parameters were measured on a sample of the crew (n = 6, 32(12) years) on one boat during one of their work shifts. In order to quantify heat production and work intensity during the time on the trawl deck, core temperature (T_core_) was measured using an ingested telemetric gastrointestinal temperature pill (Vital Sense Jonah capsule, Mini Mitter Inc, Bend) and was recorded together with heart rate (HR) using an Equivital EQ02 Life Monitor (Hidalgo, Cambridge, UK).

## Results

Seventy-seven per cent of the respondents (n = 115) reported their own health as being very good or good. 53% (n = 80) and 55 % (n = 83) had experienced stiffness and/or pain from the neck/shoulders and lower back/small of the back respectively during the last 12 months. Forty-eight per cent (n = 72) answered that they sometimes or often feel cold at work, while 39% (n = 58) answered that they have experienced a loss of feeling in their fingers/hands and 14% had suffered frostbite or cold damage. The physiological data demonstrated that during the 80(35) min work period on the trawl deck the T_core _ rose from 36.8(0.3) °C to a maximum of 37.9(0.4) °C. During the same period work above 67% of HR_max _(somewhat hard) corrected for age and upper body work were registered for long periods. HR of 149(11) BPM.min^-1 ^corresponding to 86% of HR_max _(hard) were measured for shorter periods.

## Discussion

In accordance with previous studies from Norwegian coastal fishing [1] work on trawl deck can be characterized as an intermittent activity with average levels of cardiac strain. We also registered a high occurrence of heavy cardiac strain, shown as time spent working at hard intensities. Combined with repetitive work in cold environments this may have negative effects on muscle function and fatigue [2].

## Conclusion

This study confirms that workers on deep-sea fishing vessels are exposed to unfavorable conditions such as cold and excessive strain. The questionnaire study showed that musculoskeletal problems as well as feeling cold at work, experiences of a loss of feeling, discomfort or pain in fingers, hands and feet are common. The study also demonstrates that workers are periodically exposed to high work strain, evidenced as increased core temperature and heart rate when working on the trawl deck.
